# Household exposure and animal-bite surveillance following human rabies detection in Southern Ghana

**DOI:** 10.11604/pamj.supp.2016.25.1.6200

**Published:** 2016-10-01

**Authors:** Kofi Afakye, Ernest Kenu, Kofi Mensah Nyarko, Sherry Ama Mawuko Johnson, Florence Wongnaah, George Kwame Bonsu

**Affiliations:** 1 Ghana Field Epidemiology and Laboratory Training Programme, Accra, Ghana; 2 School of Veterinary Medicine, University of Ghana, Accra, Ghana; 3 School of Public Health, University of Ghana, Accra, Ghana; 4 Ghana Health Service, Accra, Ghana; 5 Eastern Regional Hospital, Koforidua, Ghana

**Keywords:** Human-rabies, Investigation, Post-exposure prophylaxis, animal-bite surveillance, Ghana

## Abstract

**Introduction:**

Rabies remains a neglected tropical zoonotic disease with 100% case fatality rate and estimated 6,000 global mortality annually, and yet vaccine preventable. In Ghana, rabies outbreaks receive poor response. We investigated rabies in a 5-year old boy to find the source of infection, identify exposed persons for post-exposure prophylaxis and describe animal-bite surveillance in Manya-Krobo District of Ghana.

**Methods:**

We actively searched for cases and exposures by interviewing household members of the victim, schoolmates, and health professionals using WHO case definition, interview guide and checklist. We reviewed health and veterinary records and reports, and interviewed stakeholders. Descriptive data analyses were carried out and presented using tables and charts. Recorded responses were transcribed into thematic areas and analysed.

**Results:**

Child had dog-bite at the wrist, and developed hyperactivity, hydrophobia and hyperventilation 2 months post bite. He was hospitalised and died from respiratory failure day 3 after admission. Thirty-three persons were exposed to rabies infectious material. Females were 66%, age-groups 5-15yrs and 30-59 yrs were 33.3% and 39.4% respectively. A third (11/33) were category II exposure by WHO classification and were recommended for post-exposure prophylaxis. Surveillance records showed ninety-two animal-bite cases were reported for past 12 months. Half were females, and 18-59yrs age-group was 43%. Surveillance data quality was poor.

**Conclusion:**

Rabies remains a public health burden inGhana with domestic dog as reservoir of the virus and females more vulnerable to secondary exposures. Health education on rabies should be intensified, and robust animal-bite surveillance system put in place.

## Introduction

Rabies remains a neglected tropical zoonotic disease with almost 100% case fatality rate though it is vaccine preventable [1]. WHO estimated 6,000 global rabies deaths in 2013 out of which 45% occurs in Africa. Dogs constitute a major reservoir of the virus in developing countries and account for 90% of all exposures [2]. About 40% of all dog-bite cases which result in deaths from rabies occur in children below the age of 15 years. The rabies virus becomes present in the saliva two weeks prior to the appearance of clinical signs[3, 4]. Instances where viruses were seen in saliva 3-11 days before onset of clinical signs have been documented. Transmission is mainly through contact with infective saliva, tears or nervous tissue. This could be as a result of a bite, scratch or organ transplant [5]. Timely management of dog bite wound and administration of human rabies vaccine to an exposed person effectively prevent mortalities from rabies [6]. In rabies endemic countries where domestic dog constitutes the major reservoir of the rabies virus, all dog bites are considered potentially rabid and therefore post exposure prophylaxis should be initiated immediately [7]. The continuous occurrence of rabies in an area may be partly due to human demographic and behavioural changes with corresponding changes in dog or wildlife ecology. The rate of change in human population is directly related to that of dog population [8], and therefore increasing animal rabies presents an increased risk of exposures in humans. Human rabies cases continue to occur not only due to vaccination failures, but also because of unreported cases and inadequate investigations into exposures for appropriate actions.

In Ghana, dog rabies has been increasing since 1997 when the government stopped sponsoring free dog vaccination against rabies. However, reporting rates of dog-bites and rabies cases in the country are low. Where they are reported, inadequate investigation is conducted. Laboratory confirmations are sometimes carried out in the dog but not in human victims. Investigations are centred on the biting dogs and their victims, and occasionally on the victims only. Therefore, persons with potential exposures to both the infectious dog and the infected humans are often neglected. Owing to these, there is inadequate knowledge and data on the strains of rabies virus that are circulating in the dog and human populations. Additionally, evidence-based rabies management and control strategies are lacking. On the 25th September, 2012, a previously healthy 5-year old pupil from Manya-Krobo District in Eastern Region of Ghana was seen twisting his lips intermittently, showing hyperactivity and hydrophobia, two months after a dog-bite. On the 27th September, he was sent to the district hospital and later sent to referral hospital within 24 hours of admission. On the 29th September, he died from clinical rabies. Due to the fact that other persons and animals could have had contact with the victim and the dog, public health concerns were raised. We investigated to establish the source of infection, identify persons with potential physical contact with infectious materials, recommend for provision of PEP, and to establish control measures.

## Methods

We investigated rabies in a 5-year old boy in Manya-Krobo District of Ghana in November, 2012. The investigations were four fold namely: 1. Case investigation, 2. Public health investigation, 3. Animal exposure investigation, and 4. Review of health and veterinary facility records.

**Case investigation:** We interviewed the family members of the child to describe the patient, clinical symptoms while at home, when and how these symptoms started, and the treatments and actions taken at home. Further interviews were conducted with health staff at the district and referral hospitals on the clinical manifestations while at the hospitals, laboratory analysis conducted, the type of treatments given to the patient and the outcome of these actions. These interviews were conducted with an interview guide and checklist.

### Public health investigation

We carried out active case search for new rabies cases that were linked to the index case and exposures among households in the compound where child lived, playmates, teacher and classmates from the school the child attended, and the staff at both district and referral hospitals using case definition, checklist and interview guide. A case was defined as a person presenting with an acute neurologic syndrome (encephalitis) dominated by forms of hyperactivity or paralytic syndromes, progressing towards coma and death, usually by respiratory failure, within 7-10 days after the first symptom if no intensive care was instituted in the study area. Probable exposure was defined as a person who had close contact (usually a bite, scratch or saliva) with a person or an animal displaying clinical signs consistent with rabies at time of the exposure, or within 10 days following exposure in the study area; and an Exposed was a person who has had close contact (usually a bite, scratch or saliva) with a laboratory-confirmed rabid human or animal [9]. Risk among those exposed were assessed according to the WHO categories of contact, and recommendations for PEP were made for those with reasonable level of risk [7].

### Animal exposures investigation

We interviewed the victim’s caretaker on the incidence of the bite. The dog’s owner and co-tenants in the residence of the dog were as well interviewed to identify any person with potential physical contact with the dog or infectious material from it. This action was aimed at identifying any other dog or mammal that had played with, fought with, or been bitten by the dog under study. Risk levels of contact with the dog were assessed and appropriate action and PEP recommended.

### Review of records from health and veterinary facilities

Two Health facilities and a Veterinary clinic in the district were visited for record review to identify dog-bite cases and human exposure to rabies in the district, and to establish possible link with the case under investigation. Stakeholders were interviewed using a guide. Data were entered into Microsoft Excel and descriptive analysis was carried out using frequencies, and these were presented in tables and charts. Recorded responses were transcribed into thematic areas for further analysis.

### Ethical considerations

The study was considered as emergency response to a disease outbreak of public health importance by the School of Public Health and Eastern Regional Health Directorate. Approval was sought from Regional Health Directorate and the Regional Veterinary Office. We sought for consent from the individuals that were interviewed. Confidentiality, storage and security of the data and information collected were assured.

## Results

### Case investigation

On the 25th September, 2012, a previously healthy 5-year old pupil at Atua Junction in Manya-Krobo District, started showing strange behaviour of intermittent lip twisting at home. He had progressive nausea, anorexia, short breaths, hyperactivity and hydrophobia. The child complained of difficulty in breathing, painful throat and chills for the past 2 days. On the 27 th September, he was sent to a district hospital looking restless and breathless. There was nasal discharge and chest was in-drawing. He was afebrile, jaundiced, and had sunken eyes. Laboratory investigation of the blood sample showed absence of malaria parasites, and the results for the blood chemistries were as shown in Table 1.

**Table 1:** Results of Blood Chemistry of 5-year old boy bitten by a dog two months earlier, Manya-Krobo district, September, 2012

Blood Indices	Value	Blood Indices	Value
White Blood Cells	+ 17.9x10^3^/µL	Platelets	393x10^3^/ µL
Red Blood Cell	5.44x10^6^/ µL	Lymphocytes %	30.9%
Haemoglobin	12.9g/dL	Mid Cells %	1.3%
Haematocrit	36.1%	Neutrophils %	67.8%
Mean Cell Volume	-66.4fL	Lymphocytes #	5.5x10^3^/µL
Mean Cell Haemoglobin	-23.7ps	Mid Cells #	0.2 x10^3^/µL
Mean Cell Haem. Conc.	35.7g/dL	Neutrophils #	12.2 x10^3^/µL

Based on the signs exhibited by the child, pneumonia was suspected and he was admitted. Upon admission, the child was seen agitated and hyperactive. He refused to lie on the allocated bed and started jumping around. He frequently visited the urinal. When he was being medicated, he turned away from the drinking water upon seeing it. Further interrogation with the caretaker revealed that a dog had bitten the child about two months earlier and the dog was destroyed by the owner. On the same day after the bite, a co-tenant who was a health worker bought anti-tetanus serum from the pharmacy and injected the child at home. He was not sent to any health facility, and no other medication was given to him until symptoms showed up two days prior to being sent to the district hospital. The caretaker admitted that the child had been hydrophobic for three days preceding hospitalisation. Rabies was then suspected and the child was immediately referred to a higher hospital within 24 hours of admission. The referral was on account of hydrophobia and aggressive behaviour after a bite from stray dog. On the 27th September at 17.17 GMT, the child was seen at the referral hospital. He was aggressive and anxious, with intermittent hyperventilation. There was hydrophobia and he appeared very agitated. At 17.55 GMT, the child was restrained in bed by two of his relatives and was given intramuscular 300mg phenobarbitone. After the injection he became calm. This was followed by intravenous 40mg phenobarbitone every 12 hours and 1.2 litres intravenous fluids at 50mls/hr. He was closely observed. Temperature was 36.3°C; colour and hydration were normal; respiratory rate was 28 breaths per minute; pulse was 100 beats per minute. He became very restless throughout the night. On the 28th September, the boy was still restless. Respiratory rate was 22 breaths per minute and there was crepitation. Aspiration pneumonia was suspected. He was given 10mg Chlorpromezine intramusculary every 6 hours; blood pressure was monitored every 2 hours; intravenous fluids, Amoksiklav and phenobarbitone were given. On the 29th September, the boy was child was restless and could not sleep during the night. However, he was conscious and communicating. Medication for previous day was repeated. At 22.05 GMT the child stopped breathing. There was no cardio-pulmonary activity; peripheral pulses were absent, and pupils were dilated. He was pronounced clinically dead.

### Public health investigation

On 21st October, 2012 the New Juaben Municipal Health Directorate notified the Eastern Regional Public Health Directorate that human rabies has been detected at the Regional Hospital. Initial investigation by the New Juaben Municipal Health Directorate indicated that the patient died of clinically-confirmed rabies on the 29th September, 2012. Further investigation by the team revealed that symptoms were seen on 25th September, 2012. Humans can begin to shed rabies virus in saliva and tears 2 weeks before onset of symptoms [10]. The patient was therefore considered to be potentially infectious during September 11th - 29th, the period from 2 weeks before onset of symptoms until his death. The victim travelled from Manya-Krobo District to Kwahu West District between 26th July and 4th September 2012 to spend the school long vacation holidays. Interrogations with the class teacher indicated that the last time the child attended school was 6 th September. During these periods he was not potentially infectious. The case and exposure search showed that total number of persons that came into contact with the child and the dog were 33: two thirds of these were classified as category I exposures, a third as category II and none as Category III (Table 2).

**Table 2:** Characteristics of persons potentially exposed to rabies infectious material at Manya-Krobo district, 2012

Place exposure occurred	*Category I Exposure		#Category II Exposure		Total, n (%)
	Male	Female	Male	Female	
Child’s Home	5	2	3	3	13 (39.3)
District Hospital	1	6	0	1	8 (24.3)
Regional Hospital	1	5	0	1	7 (21.2)
Dog’s Home	1	1	1	2	5 (15.2)
Child’s School	0	0	0	0	0 (0)
**Age Group exposure occurred**					
<5	1	0	0	0	1 (3.0)
5-15	5	1	3	2	11 (33.3)
16-30	0	6	0	1	7 (21.2)
30-59	2	7	1	3	13 (39.5)
>59	0	0	0	1	1 (3.0)
Total, n (%)	8(24.3)	14(42.4)	4(12.1)	7(21.2)	33 (100)
	22 (66.7)		11 (33.3)		

* Touching, feeding of animals or licks on intact skin (no exposure therefore no prophylaxis if history reliable)

# Minor scratches or abrasions without bleeding or and nibbling of uncovered skin (use vaccine alone)

Twenty-eight persons came into contact with the child while he was infectious between September 11th and 29th 2012. Almost half of this occurred among the households, and the rest at the Hospitals. However, 46% (6/13) of the home exposures were category II exposures as against 13.3% (2/15) at the Hospitals. They were recommended for PEP. Out of the total exposures females constituted 66% whereas within the households they formed 50%. The most exposed age-group to the child was 30-59 year old (43%), followed by persons between 5 and 15 years old (29%). None of the exposed persons developed rabies.

### Investigation of animal exposures

During the last week of July 2012, the child followed his 15 year old sister who had gone to a petty shop across the street. At the third compound from their house, the child was running to catch up with the sister when the dog attacked and bit him at the wrist. Five other persons had contact with the dog where two females and a male were classified as category II exposures and were therefore indicated for PEP (Table 2).

### Review of records from health and veterinary facilities

**Figure 1:**
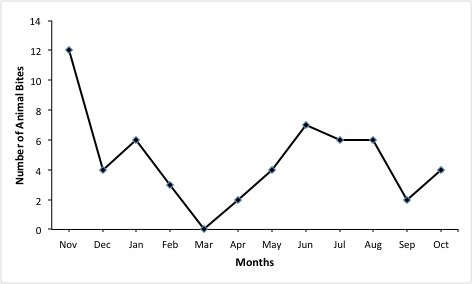
Reported animal-bites at government Hospital between Nov. 2011 and Oct. 2012, Manya Krobo District

A total of 92 animal-bite cases were reported for the period November 2011-October 2012 from two health facilities in the district: a government-owned hospital 56(61%) and a private-owned hospital 36(39%) (Table 3). Proportions of males and females with animal-bites were equal for the district. However, unlike the private-owned hospital, males were more than females at the government-owned hospital. Generally, age-group 18-35 years had the highest proportion of animal-bite cases. Among the males, age-group 5-14 years was the most bitten with 15(33%) cases, whereas age-group 18-35 years recorded the highest number of 17(40%) bites among the females. The trend of the animal-bite cases at the government hospital showed peaks in November, January, with a high plateau in June, July and August (Figure 1). The deepest trough was recorded in March. The District Veterinary Clinic reported 11 dog-bite cases for the same period. These did not represent additional cases since they were captured at the health facilities as animal-bite cases. Out of the 11 cases, six (55%) were reported from the government hospital, two (18%) from mission hospital, another two (18%) from outside the district and one used veterinary clinic as first point of reporting. Six (55%) were males but their ages were not recorded. Samples from the 11 dogs reported at the veterinary clinic were taken to the laboratory to test for rabies. Out of the 11 dog-bites 1 (9%), which occurred on 27 th June, 2012 was laboratory confirmed positive after the dog was put under quarantine and naturally died three days later. An eight year old boy was a victim to this dog-bite case and was given PEP. This victim did not develop rabies. Outcome information on other victims were not available. The type of data captured on the case-based forms were inadequate and incomplete for meaningful analysis. Again, there was no meaningful linkage between the animal bites captured at the health facilities and those at the veterinary department. Collaboration between these two bodies was weak.

**Table 3:** Number of reported animal bites by sex and age group, Manya-Krobo District, 2011/2012 (N=92)

Type of Facility	Sex	Age Group						Total, n (%)
		<5yrs	5-14yrs	15-17yrs	18-34yrs	35-59yrs	>59yrs	
Public Hospital	Male	1	11	4	4	1	3	24 (26)
	Female	2	6	1	13	5	1	32 (35)
Private Hospital	Male	5	4	2	8	3	0	22 (24)
	Female	1	4	0	4	2	7	14 (15)
Total, n (%)		9 (10)	25 (27)	7 (8)	29 (31)	11 (12)	11 (12)	92 (100)

## Discussion

Rabies in humans is a potential public health problem in Ghana but the severity is not perceived due to underreporting and improper documentation. Animal-bites are the main source of transmission of rabies virus in most African countries [2], and can serve as a valuable source of epidemiological data [11]. This study investigated rabies case to identify the source of infection and persons potentially exposed to infective materials, and to describe animal-bite surveillance in Manya-Krobo District. Findings indicated that the human rabies case was epidemiologically linked to the dog-bite sustained by the victim as the likely source of the infection. One third of persons potentially exposed to infective materials met the category II exposure according to WHO classification. Data quality and completeness were major challenges in the animal-bite surveillance system. The results of this study indicated that hyperactivity, hydrophobia, and hyperventilation were prominent in the neurologic syndrome exhibited by the child. These findings are consistent with what have been described elsewhere [12]. The findings describe furious type of rabies more than the paralytic form. These information are useful for medical interventions since there has been an instance of symptomatic treatment of clinical rabies with favourable outcomes [13]. The incidence of dog-bite was confirmed during our interviews. However, no laboratory confirmation was obtained from the dog because it was not available as at the time of the investigation since the owner destroyed it soon after the bite, and no sample was taken. This does not exclude the fact that the dog was the source of the infection [14]. Considering the part of the body that was bitten, the period between the bite and the manifestation of neurologic syndrome, and the subsequent progression and outcome of the disease, a plausible link can be established between the dog-bite and the development of rabies in the victim. This finding is in agreement with the results of other studies which incriminated domestic dog as major source of rabies virus in developing countries [2, 11, 15] This could be explained by the fact that neither dog population control nor measures to control rabies in the domestic dog were being taken. Studies indicated that the virus is maintained in the domestic dog when their population densities grow to a critical level [16]. As dog populations grow, dog to human ratio increases and spatial proximity between the two populations reduces in size.

A third of those that were exposed to infectious materials of the child and dog were indicated for PEP in our study. This is in contrast with other studies where 18% [15] and 12% [14] were recorded. The higher proportion in this study could have resulted from the relatively fewer number of people that were contacted for interview, which made the denominator to be low. This happened since the victim in this study did not embark on travelling and other movements during the infective stage, which would have brought him in contact with many people. This implies that people whose daily activities involve a lot of movement are likely to spread the virus should they become infected. Contact investigations for PEP in such instances may be frustrating and should therefore be carried out cautiously to capture all suspected exposed persons. It is indicated that administration of PEP to persons exposed to rabies virus should not be considered as medical emergency but rather as medical urgency [5]. Our findings showed that the animal-bite surveillance system has challenges in data quality and completeness. This is in contrast with what Obonyo and his colleagues found [15]. Good and adequate data is crucial for evaluation of any disease surveillance system simply because accurate results are obtained for appropriate actions to be taken. In our study, the case-based forms used at the district hospitals were designed to capture very few data on the animal-bite victim and his/her health condition, and no data at all on the animal culprit. The incomplete data at the veterinary facility added to the challenge. This implies that using these data in surveillance data analysis for decision and action will not give meaningful results. The weak collaboration between the health and the veterinary departments on issues of zoonoses corroborates the findings in Kenya [15] but contrasts what was found in USA [14]. This may be due to failure of these important institutions to appreciate their complementary roles in dealing with health issues involving humans and animals. In such circumstances, effects of zoonotic disease outbreaks become severe due to improper management.

## Conclusion

Rabies continues to pose public health challenges in Ghana, and domestic dog is still reported as the major reservoir of the virus in the country. Females and children are more vulnerable to animal-bites and secondary exposures especially during school vacation holidays. There is weak collaboration between human and animal health departments resulting in weak animal-bite surveillance system. Health education on zoonoses should be intensified, especially among parents and children so that all victims of dog-bites would receive PEP. Well-equipped systems should be put in place by the human and animal health sectors to ensure adequate surveillance, timely emergency response, prevention and control of rabies through the one-health approach.
